
JOURNAL INFORMATION
==============================
NLM Title Abbreviation: ScientificWorldJournal
Journal ID: ScientificWorldJournal
NLM Journal ID: 101131163
Journal ID: TSWJ

The Scientific World Journal

ISSN: 2356-6140
EISSN: 1537-744X
Publisher: Wiley

ARTICLE INFORMATION
==============================
PMCID: PMC6083961
PMID: 30147557
DOI: 10.1100/tsw.2001.117
Article ID: 318359
Article version: 1
Subjects: Short Report

Caloric Intake, Oxidative Stress and Aging

Lee Cheol Koo
Prolla Tomas A.
Weindruch Richard *
Departments of Genetics, Medical Genetics, and Medicine, University of Wisconsin, Madison WI 53706, USA
*Corresponding author.
Publication date: 2001
Electronic publication date: 2001 May 1
Volume: 1
First page: 85
Last page: 85
Copyright: Copyright © 2001 Cheol Koo Lee et al.
Copyright year: 2001
License: This is an open access article distributed under the Creative Commons Attribution License, which permits unrestricted use, distribution, and reproduction in any medium, provided the original work is properly cited.
License URL: https://creativecommons.org/licenses/by/3.0/

Funding: National Institutes of Health P01 AG11915 National Institutes of Health R01 AG18922 National Institutes of Health R01 CA78723

==============================
Miami *Nature Biotechnology* Short Reports
*TheScientificWorld* (2001) 1(S3), 85SR
ISSN 1532-2246; DOI 10.1100.TSW.2001.117

### CALORIC INTAKE, OXIDATIVE STRESS AND AGING

Cheol Koo Lee, Tomas A. Prolla, and Richard Weindruch*

Departments of Genetics, Medical Genetics, and Medicine,
University of Wisconsin, Madison WI 53706 USA

**INTRODUCTION.** To examine the molecular events associated with aging in mammals, we employed oligonucleotide-based arrays to define the transcriptional response to the aging process in mouse gastrocnemius muscle (1) and in two regions of the brain (cerebellum, neocortex) (2). We also studied the influence of retarded mammalian aging induced by caloric restriction (CR) (3, 4) on transcriptional patterns in these tissues. Our choice of tissues was guided by the fact that skeletal muscle and the brain are primarily composed of long-lived, high oxygen-consuming postmitotic cells, a feature shared with another critical aging target (the heart).

**METHOD.** Male C57BL/6 mice were fed either a normal caloric intake or subjected to CR (a 26% reduction in caloric intake). To investigate the influence of age, 5-month-old mice ($n = 3$) were compared to 30-month-old mice ($n = 3$) by generating a total of nine pairwise comparisons of the fold changes in gene expression. The average fold change and standard error was computed. To study the influence of CR, 30-month old controls were compared in this same manner to 30-month old mice on CR since 2-months of age. mRNA populations from individual animals were used to generate cRNA, which was then hybridized to individual oligonucleotide arrays (Affymetrix, Santa Clara, CA) to provide data on 6347 genes. The DNA chips were read at a resolution of 6 μm with a Hewlitt-Packard GeneArray Scanner.

**RESULTS.** In the gastrocnemius, aging resulted in gene expression profile indicative of a marked stress response and lower expression of metabolic and biosynthetic genes. Most alterations were either completely or partially prevented by CR, the only intervention known to retard aging in mammals. Transcriptional patterns of calorie-restricted animals suggest that CR retards the aging process by causing a metabolic shift toward increased protein turnover and decreased macromolecular damage. In both regions of the brain, aging resulted in a gene expression profile suggestive of a marked inflammatory response, oxidative stress, and reduced neuronal plasticity and neurotrophic support. At the transcriptional level, brain aging in mice displays striking parallels with human neurodegenerative disorders. In the brain, CR selectively attenuated the age-associated induction of genes encoding inflammatory and stress responses.

**DISCUSSION.** Our observations suggest that specific gene expression patterns are associated with the aging process of individual organs, and that CR can prevent or retard the establishment of many such gene expression alterations. A common feature observed in tissues from old, normally fed mice was the induction of a stress response consistent with a state of increased oxidative stress. This change was opposed by CR. The use of DNA microarrays generates panels of hundreds of transcriptional biomarkers of molecular aging. Accordingly, this approach provides a new tool to measure biological age on a tissue-specific basis and to evaluate, at the molecular level, the efficacy of interventions designed to retard the aging process. We are currently using oligonucleotide arrays to investigate the influence of CR on aging in rhesus monkeys.

**ACKNOWLEDGEMENT.** Supported by NIH grants P01 AG11915 (R.W.), R01 AG18922 (R.W.) and R01 CA78723 (T.A.P.).

**REFERENCES.**

1. Lee, C.-K., Klopp, R.G., Weindruch, R., and Prolla, T.A. (1999) Science 285, 1390-1393
2. Lee, C.-K., Weindruch, R., and Prolla, T.A. (2000) Gene expression profile of the ageing brain. Nat. Genet. 25, 294-297
3. Weindruch, R. and Walford, R.L. (1988) J. Gerontol. 43, B40-42
4. Weindruch, R. (1996) Sci. Am. 274, 46-52
